# Basal metabolic state governs AIF-dependent growth support in pancreatic cancer cells

**DOI:** 10.1186/s12885-016-2320-3

**Published:** 2016-04-23

**Authors:** Andrew J. Scott, Amanda S. Wilkinson, John C. Wilkinson

**Affiliations:** Department of Chemistry and Biochemistry, North Dakota State University, Dept. 2710, P.O. Box 6050, Fargo, ND 58108-6050 USA

**Keywords:** Mitochondria, Cell death, Glycolysis, Oxidative phosphorylation

## Abstract

**Background:**

Apoptosis-inducing factor (AIF), named for its involvement in cell death pathways, is a mitochondrial protein that regulates metabolic homeostasis. In addition to supporting the survival of healthy cells, AIF also plays a contributory role to the development of cancer through its enzymatic activity, and we have previously shown that AIF preferentially supports advanced-stage prostate cancer cells. Here we further evaluated the role of AIF in tumorigenesis by exploring its function in pancreatic cancer, a disease setting that most often presents at an advanced stage by the time of diagnosis.

**Methods:**

A bioinformatics approach was first employed to investigate AIF mRNA transcript levels in pancreatic tumor specimens vs. normal tissues. AIF-deficient pancreatic cancer cell lines were then established via lentiviral infection. Immunoblot analysis was used to determine relative protein quantities within cells. Cell viability was measured by flow cytometry; in vitro and Matrigel™ growth/survival using Coulter™ counting and phase contrast microscopy; and glucose consumption in the absence and presence of Matrigel™ using spectrophotometric methods.

**Results:**

Archival gene expression data revealed a modest elevation of AIF transcript levels in subsets of pancreatic tumor specimens, suggesting a possible role in disease progression. AIF expression was then suppressed in a panel of five pancreatic cancer cell lines that display diverse metabolic phenotypes. AIF ablation selectively crippled the growth of cells in vitro in a manner that directly correlated with the loss of mitochondrial respiratory chain subunits and altered glucose metabolism, and these effects were exacerbated in the presence of Matrigel™ substrate. This suggests a critical metabolic role for AIF to pancreatic tumorigenesis, while the spectrum of sensitivities to AIF ablation depends on basal cellular metabolic phenotypes.

**Conclusions:**

Altogether these data indicate that AIF supports the growth and survival of metabolically defined pancreatic cancer cells and that this metabolic function may derive from a novel mechanism so far undocumented in other cancer types.

## Background

Apoptosis-inducing factor (AIF) is a mitochondrial flavoprotein discovered and named for its involvement in caspase-independent cell death, and the mechanisms through which AIF mediates cellular toxicity have been largely defined [1–11]. Distinct from its death role, AIF also possesses an intrinsic NADH oxidase activity that is linked to control of mitochondrial structure and function [12, 13]. A complete picture of how AIF promotes mitochondrial homeostasis remains elusive, but it has become increasingly clear that through its enzymatic activity AIF has a primary role in maintaining cell survival. Inactivation of the AIF gene in mice causes embryonic lethality [14, 15], whereas the Harlequin mouse model exhibits severe and progressive neurodegeneration as a consequence of decreased AIF protein expression (>80 %) in all tissues [16]. Tissue-specific AIF deletion studies demonstrated a series of physiological defects including skeletal muscle atrophy and dilated cardiomyopathy resulting from severe mitochondrial dysfunction and loss of cristae structure [17, 18]. More recent studies in humans have identified AIF mutations that lead to respiratory chain malfunction with a spectrum of clinical manifestations including mitochondrial encephalomyopathy [19], prenatal ventriculomegaly [20], and Cowchock syndrome [21]. The AIF-deficient phenotype is associated with concomitant depletions of mitochondrial respiratory chain subunits and subsequent impairment of oxidative phosphorylation [22], in part due to a resulting defective co-translational import system regulated by the AIF-interacting protein CHCHD4/MIA40 [23, 24]. It has been proposed that AIF functions in vivo as a metabolic sensor [25], supported by its contributory roles to disorders involving metabolic dysregulation including obesity, diabetes, and cancer [26–28]. While largely descriptive, these studies altogether illustrate a role for AIF as a critical regulator of cellular metabolism.

The pro-survival activity of AIF and its role in controlling metabolic homeostasis in healthy cells is well positioned to be exploited by cancer cells in order to promote growth, invasiveness, and chemoresistance [28, 29]. Indeed, increased AIF protein levels have been observed in esophageal, skin, colorectal, gastric, lymphatic, and prostate cancers [28, 30–35], and in colorectal cancer increased AIF levels elevate the general cellular oxidative state to protect cells from chemical stress [29]. Furthermore, we have shown that in prostate cancer the NADH oxidase activity of AIF promotes a metabolic state that supports growth and invasiveness in a manner specific to cells that have achieved advanced status [28]. Given our findings that AIF preferentially supports metabolism benefitting the aggressiveness of advanced-stage prostate cancer cells, we questioned whether AIF also contributes to pancreatic ductal adenocarcinoma (PDAC), a disease which almost always reaches an advanced stage before diagnosis [36]. Unfortunately for patient prognosis, late detection of PDAC makes it one of the most lethal cancers with a grim 5-year survival rate of 6 % [37]. As tumors progress and achieve advanced stages, reliance upon specific metabolic pathways for growth and survival increases substantially while cells become vulnerable to death by metabolic disruption, a trait known as metabolism addiction. As one of the most metabolically driven forms of cancer, mitochondrial function is frequently critical to pancreatic tumorigenesis [38, 39], leading us to hypothesize that AIF’s metabolic function supports the growth and survival of PDAC cells.

In this study we identified AIF as a major contributor to the growth-promoting metabolic state of pancreatic tumor cells. The contribution of AIF to PDAC metabolism in our panel of cell lines was directly related to their basal metabolic preferences. While cells that use both glycolysis and mitochondrial energy metabolism rely on AIF for survival, those that rely only upon glycolysis cannot benefit from AIF’s metabolic activity. Through a mechanism that appears distinct from its function in prostate cancer, we found that AIF facilitates a metabolic balance that maintains survival, a role that potentially extends to normal tissues and may explain the selective sensitivity to AIF suppression among cell types. Altogether our findings suggest that AIF is a significant support molecule to the development and progression of some pancreatic cancers and therefore represents a promising new target for therapeutic development.

## Methods

### Materials

MEM, DMEM, RPMI 1640, DMEM/F12, GlutaMAX, horse serum, insulin, transferrin, epidermal growth factor, trypsin, 4–12 % bis-tris polyacrylamide gels, and nitrocellulose membranes were obtained from Life Technologies; fetal bovine serum (FBS), phosphate buffered saline (PBS), and Pierce ECL 2 Western Blotting Substrate were from Thermo Scientific; QuantiChrom™ Glucose Assay Kit was from BioAssay Systems; Matrigel™ was from BD Biosciences; Matrigel Recovery Solution was from Corning; protease inhibitor tablets were from Roche Applied Science; all other materials were from Sigma. Antibodies were obtained as follows: anti-AIF (Santa Cruz Biotechnology, sc-13116), anti-complex I 39 kDa (Life Technologies, 459100), anti-complex I 20 kDa (Life Technologies, 459210), anti-complex I 17 kDa (Life Technologies, A21359), anti-COX IV (Life Technologies, A21347) anti-β-actin (Sigma, A5316), and peroxidase-conjugated anti-mouse (Amersham Biosciences, NA931V).

### Oncomine data analysis

Data sets examining AIF mRNA expression in pancreatic tumors versus normal pancreatic tissue from 7 studies [40–46] were analyzed using Oncomine [47]. Statistical calculations and normalization techniques are given by the Oncomine website (http://www.oncomine.org).

### Cell culture

PANC-1, BxPC-3, HPAF-II, HPAC, and MIA PaCa-2 cells were from ATCC (kind gift of Dr. Sanku Mallik, NDSU). HEK293T cells were as described [28]. Cells were grown in an atmosphere of 95 % air and 5 % CO_2_ at 37 °C. All media was supplemented with 2 mM GlutaMAX. Cell lines were grown and cultured with the following media formulations: HEK293T and PANC-1 cells in DMEM supplemented with 10 % FBS; MIA PaCa-2 in DMEM supplemented with 10 % FBS and 2.5 % horse serum; BxPC-3 in RPMI 1640 supplemented with 10 % FBS; HPAF-II in MEM supplemented with 10 % FBS; and HPAC in a 1:1 mixture of DMEM and Ham’s F12 medium supplemented with 5 % FBS, 2 μg/mL insulin, 5 μg/mL transferrin, 40 ng/mL hydrocortisone, and 10 ng/mL epidermal growth factor.

### Lentivirus production and infection

FG12-derived plasmids for targeting of AIF and LacZ by RNA interference (RNAi) have been rigorously assessed and used as described [28, 48, 49]. Lentiviral packaging plasmids pRRE, pRSV-rev, and pHCMV-G are as described [50]. RNAi plasmids and equal amounts of lentiviral packaging plasmids were transfected into HEK293T cells using the calcium phosphate precipitation method [51]. Supernatants of transfected HEK293T cultures were then filtered through 0.45-μm PVDF Millex-HV filters (Millipore) and concentrated by centrifugation at 20,000 × g for 90 min at 4 °C. Viral pellets were resuspended in PBS and then incubated overnight at 4 °C prior to use. Cell lines were then infected as described [28]. PANC-1 and MIA PaCa-2 cells were infected with lentiviruses carrying shLacZ-GFP or shAIF-GFP, after which infection was verified by fluorescence microscopy and flow cytometry with an Accuri C6 flow cytometer. BxPC-3, HPAC, and HPAF-II cells were infected with lentiviruses carrying shLacZ-puro or shAIF-puro and then selected using 2 μg/mL puromycin.

### Cell viability

Cells were seeded in replicate populations of 25,000-150,000 cells per well in 6-well plates and allowed to attach overnight. Cells were then left untreated or treated with 1 μg/mL actinomycin D, 1 μM gemcitabine, or 50 mM 2-deoxyglucose for 24–72 h. Cells were then harvested by trypsinization, washed, and resuspended in PBS containing 2 μg/mL propidium iodide. Cell viability was determined by flow cytometry.

### SDS-PAGE and immunoblotting

Cells were harvested by trypsinization, washed, and resuspended in radioimmune precipitation assay lysis buffer (PBS containing 1 % Nonidet P-40, 0.5 % sodium deoxycholate, 0.1 % sodium dodecyl sulfate, 1 mM dithiothreitol, 1 mM phenylmethanesulfonyl fluoride, and 1 protease inhibitor mixture tablet per 10 mL). Lysates were then normalized for protein content, separated by SDS-PAGE, and transferred to nitrocellulose membranes. Membranes were blocked with 5 % milk in Tris-buffered saline with 0.1 % Tween-20 and incubated with primary antibodies for 1 h at room temperature. Membranes were then washed and incubated with peroxidase-conjugated anti-mouse IgG secondary antibody for 45 min at room temperature, followed by washing and visualization using enhanced chemiluminescence with a MyECL imaging system (Thermo Scientific).

### Cell growth rate measurements

Cells were harvested by trypsinization, washed, resuspended in fresh medium, and seeded at equal densities in replicate 6-well plates. Cells were harvested and quantified by Coulter™ counting after 72 h. Fold change in growth was determined by dividing populations of AIF-deficient cells by corresponding control populations.

### Scratch assay

Cells were harvested by trypsinization, washed, resuspended in fresh media, and seeded in replicate 6-well plates. Cells were allowed to attach for 12–36 h, and a single scratch was made through the middle of each well using a P200 pipette tip [52]. Cells were immediately washed, fresh media was added, and each scratch was imaged. Cells were then incubated for 6–48 h before final assessment of scratch width. All images were captured by phase contrast microscopy using the 10× objective of a Nikon TS100F microscope equipped with a Nikon DS-Fi1 digital camera detection system and NIS Elements 4.0 software.

### Glucose consumption measurements

Cells were harvested by trypsinization, washed, resuspended in fresh medium, and seeded at equal densities in replicate 6-well plates. Cells were grown at 37 °C for 72 h. Media was then collected from each well, and total glucose was measured using the QuantiChrom™ Glucose Assay Kit (BioAssay Systems). Total cell number in each sample was determined by Coulter™ counting. To determine glucose consumed per cell, total glucose consumption per sample was divided by its corresponding cell count.

### Matrigel™ experiments

Equal volumes of cold Matrigel™ were added to each well in 24-well plates and allowed to solidify at 37 °C for 1 h. Cells were harvested, washed, resuspended in fresh medium, and seeded in equal densities on solidified Matrigel™ layers. Cells were grown in Matrigel™ for up to 21 days with media replenished following each week of growth and imaged using phase contrast microscopy as described above. Cells were then extracted from substrate with the Matrigel Recovery Solution (Corning), and glucose consumption and growth measurements were performed as described above.

## Results

### Elevation of AIF mRNA transcripts in pancreatic cancer

**Fig. 1:**
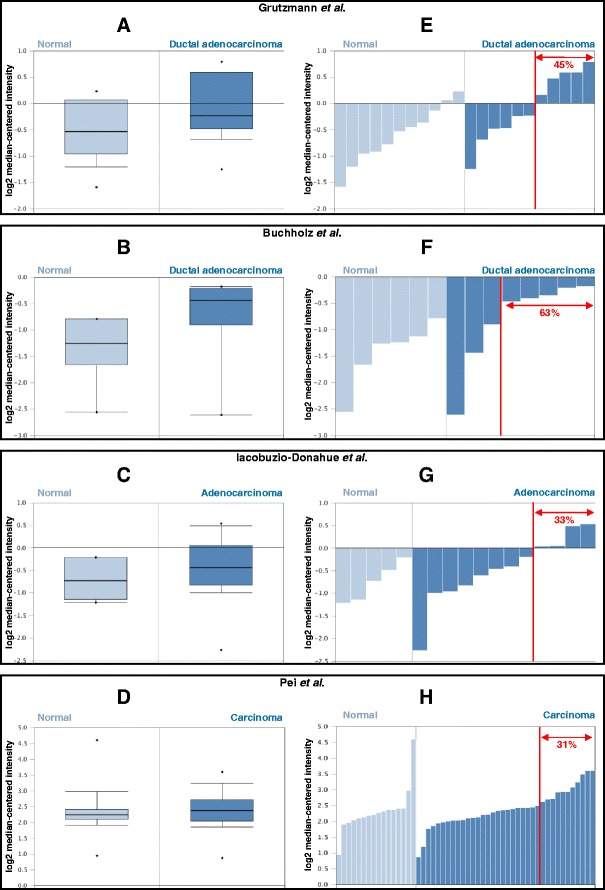
AIF expression in pancreatic cancer. Data comparing AIF mRNA transcript expression in pancreatic tumors compared to normal pancreatic tissue was retrieved from the Oncomine database and assessed as shown in representative studies [40–42, 46]. Panels **a**-**d**: Average relative AIF mRNA expression in pancreatic tumors vs. normal pancreatic tissue. Panels **e**-**h**: Relative AIF mRNA levels among individual samples within each cohort. Fractions of tumor specimens within each cohort exhibiting statistically significant AIF expression changes relative to normal tissue are indicated in *red*

To determine a potential involvement of AIF in pancreatic cancer, we began by analyzing archived expression data retrieved from the publically available cancer gene expression database Oncomine (oncomine.com). Archival data from a total of 7 data sets comparing relative AIF expression in pancreatic adenocarcinomas to normal pancreatic tissue are currently available [40–46]. When average AIF expression was compared between groups (cancer vs. normal) and for each data set, a trend towards elevated AIF transcripts in pancreatic cancer tissue was apparent (values ranging from essentially unchanged to an increase of 1.54 fold in cancer relative to normal). However, the observed increase in average expression was only statistically significant in one of the seven data sets, which indicated a 1.45-fold increase in AIF expression in pancreatic cancer tissue ([40], *p* = 0.024; for all other studies *p* = 0.068–0.956). Representative data are shown in Fig. 1a-d. At first glance these data suggested that altered AIF expression is not a global trend in pancreatic cancer, but that in a small fraction of tissue cohorts a modest (less than 2-fold) elevation of AIF expression can be observed.

To further explore AIF expression changes in these cohorts of cancer vs. normal tissues we compared individual expression data from each sample within each cohort (Figs. 1e-h). Interestingly, in 5 of the 7 data sets there appears a subtype within each cancer group that displays elevated AIF expression significantly beyond the 90 % confidence interval defined for normal tissues [40–43, 46]. This subtype represents ~36 % of the total among these five cohorts, and while elevated AIF is observed, the magnitude of this elevation remains modest (less than 3-fold relative to control tissue). These data are in close agreement with similar analyses examining AIF mRNA and protein expression in prostate cancer tissues [28]. Taken together these data suggest that while elevated AIF expression is not a global feature of pancreatic cancer, there exists a subtype of pancreatic tumors (approximately one-third of the total samples assessed) in which AIF expression is significantly elevated. That increased expression is modest likely reflects potential toxicity associated with AIF-mediated cell-killing when levels exceed a certain threshold [1].

### Establishment of AIF-deficient cell lines

**Fig. 2:**
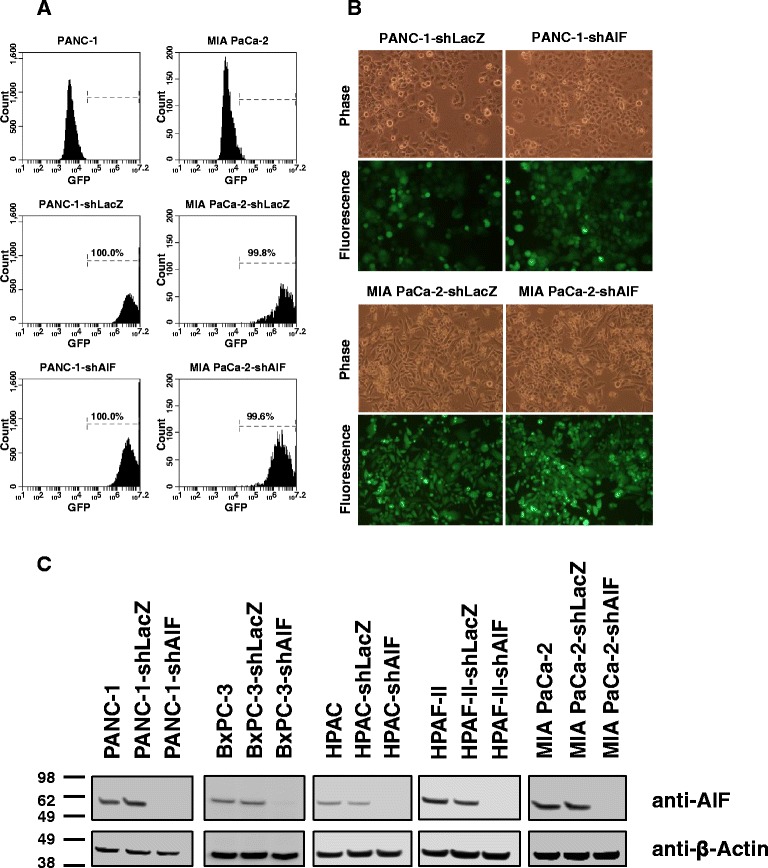
Establishment of AIF-deficient pancreatic cancer cell lines. PANC-1 and MIA PaCa-2 cells were stably infected with shRNA hairpins targeting LacZ or AIF with GFP as a selectable marker via lentiviral delivery. GFP positivity of infected cell lines was assessed by flow cytometry (Panel **a**) and fluorescence microscopy (Panel **b**). Equivalent targeting in BxPC-3, HPAC, and HPAF-II cells was achieved by puromycin selection; suppression of AIF protein expression was verified by immunoblot analysis (Panel **c**)

The above analysis of AIF gene expression data from clinically derived pancreatic cancer tissues suggested a connection between elevated AIF expression and subtypes of pancreatic tumors. In order to evaluate the role of AIF in the growth and survival of pancreatic cancer cells, we generated a panel of AIF-deficient PDAC cell lines. Given the metabolic activity of AIF in other systems, we targeted AIF in a panel of five cell types (PANC-1, BxPC-3, HPAC, HPAF-II, and MIA PaCa-2) that display diverse metabolic characteristics. Previously, metabolic phenotyping of PDAC cells was rigorously established by gene expression analysis, sensitivity to metabolic inhibitors, and metabolite profiling [53]. PANC-1 and BxPC-3 cells balance the metabolic requirements derived from glycolysis, pentose phosphate pathway, and mitochondrial energy metabolism, while HPAC cells display a pronounced bias towards lipogenic pathways; HPAF-II cells also require mitochondrial energy pathways but are more reliant upon glycolysis than those previously described, and MIA PaCa-2 cells are highly glycolytic (Table 1). Within this panel of cell lines oncogene status varies slightly: all cells except BxPC-3 express mutant KRAS, and all cells except HPAC express mutant p53 [54–60]. Silencing of AIF expression by RNAi was achieved through infection with lentiviruses harboring short-hairpin RNA (shRNA) sequences targeting either AIF (shAIF) or LacZ (shLacZ) as a control. PANC-1 and MIA PaCa-2 cells were infected with lentiviruses that carried GFP expression cassettes which served to confirm stable integration and subsequent knockdown of targeted genes. Success of lentiviral infection was evaluated by observation of at least 95 % GFP positivity using fluorescence microscopy and flow cytometry (Fig. 2a, b). Due to lower infection efficiencies, BxPC-3, HPAC, and HPAF-II cells were infected with lentiviruses carrying the puromycin N-acetyl transferase gene as a selectable marker instead of GFP, and stably infected cells were derived by treatment with puromycin. To control for differences between lentiviruses bearing puromycin resistance vs. GFP, we additionally established PANC-1 cells via puromycin selection that were indistinguishable from GFP-infected cells in all assays performed (data not shown). To determine the extent of AIF protein ablation in our cell line panel, immunoblot analysis was employed, which demonstrated AIF knockdown levels greater than 95 % in all cases when compared to either uninfected cells or shLacZ negative controls (Fig. 2c).

**Table 1:** AIF dependence is related to metabolic phenotype [53] and sensitivity to glycolytic inhibition in pancreatic cancer cells

	Relative metabolic phenotype [53]		Sensitivity to glycolytic disruption	Sensitivity to AIF ablation
	Glycolysis/PPP	Fatty acid/ OXPHOS		
PANC-1	Moderate	Moderate	Insensitive	Sensitive
BxPC-3	Moderate	Moderate	Insensitive	Sensitive
HPAC	Low	High	Insensitive	Sensitive
HPAF-II	High	Moderate	Moderately sensitive	Moderately sensitive
MIA PaCa-2	High	Low	Sensitive	Insensitive

### AIF ablation does not affect chemical death induction in pancreatic cancer cells

**Fig. 3:**
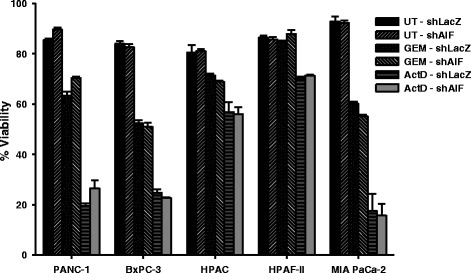
AIF ablation does not impact chemical death induction in pancreatic cancer cells. Equal numbers of cells were harvested, washed, and allowed to attach overnight. Cells were then left untreated (UT) or treated with 1 μg/mL actinomycin D (ActD) for 24 h or 1 μM gemcitabine (GEM) for 48–72 h. Cell viability was then determined by propidium iodide staining and flow cytometry. Data are shown as average ± standard deviation

In various cell types, AIF has been shown to promote both death induction and survival in response to toxic chemical triggers. To determine the role of AIF in regulating cell death in pancreatic cancer, we employed two death-inducing agents with distinct mechanisms of action: actinomycin D, an inhibitor of protein synthesis; and gemcitabine, a nucleoside analog and the first-line treatment for PDAC [61, 62]. Following treatment viability was measured by propidium iodide staining and flow cytometry. When compared to controls, AIF-deficient cell lines showed neither increased resistance (to actinomycin D) nor increased sensitivity (to gemcitabine) following treatment (Fig. 3). Similar results were obtained by treatment with etoposide, MNNG, arsenic trioxide, menadione, and hydrogen peroxide (data not shown). These data demonstrate that AIF does not play a significant regulatory role in the promotion of cell death in pancreatic cancer cells, and is consistent with previous studies evaluating AIF-mediated cell death in prostate cancer [28].

### AIF selectively supports the growth and migration of pancreatic cancer cells

**Fig. 4:**
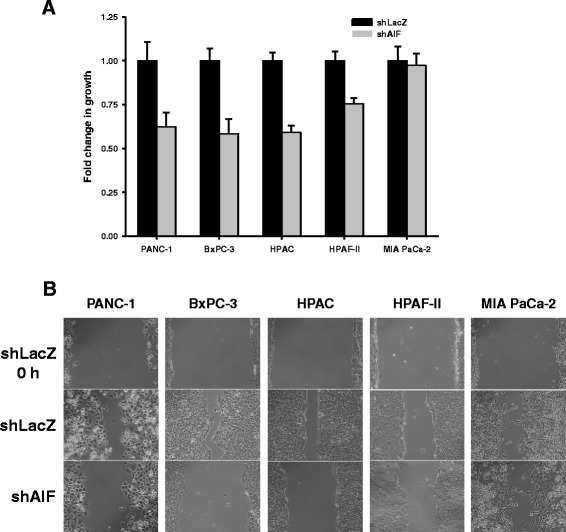
Contribution of AIF to the growth and migration of pancreatic cancer cells in vitro. *Growth assay* (Panel **a**): cells were plated in equal densities in replicate, harvested, and quantified by Coulter™ counting after 72 h of growth. Data are shown as average ± standard deviation. *Scratch assay* (Panel **b**): high densities of cells were seeded in replicate wells and allowed to attach for 12–36 h. A single scratch was made through the middle of each well, and width was assessed at 0 h (all cell lines), 6 h (HPAC), 10 h (HPAF-II), 24 h (PANC-1), and 48 h (BxPC-3 and MIA PaCa-2). Representative images are shown (Panel **b**); all images were captured at 10× magnification

In order to determine whether AIF ablation impacts the rate of proliferation of pancreatic cancer cells, we measured the growth of AIF-deficient cells in vitro. Equal populations of cells were seeded in fresh media and allowed to proliferate for 72 h before quantification by Coulter™ counting. Notably, 4 of the 5 cell lines (PANC-1, BxPC-3, HPAC, and HPAF-II) showed a reduction in growth rate following ablation of AIF. After 3 days of proliferation, AIF-deficient PANC-1, BxPC-3, and HPAC cells exhibited only ~60 % of growth compared to shLacZ controls. AIF ablation resulted in a more modest reduction in proliferation rate in HPAF-II cells (~75 %), while growth was unaffected in MIA PaCa-2 cells (Fig. 4a). These results are distinct from our previous observations: in prostate cancer the growth rates of cells are largely unaffected by AIF ablation under nutrient-rich conditions in vitro, and it is not until exposure to Matrigel™ or growth stress in vivo that AIF-deficient prostate cancer cells exhibit substantial reductions in growth. This suggests that AIF is either more important in advanced PDAC vs. prostate cancer, and/or functions via alternative/additional mechanisms.

To further define the role of AIF in controlling the aggressiveness of pancreatic tumor cells, we next assessed the migration of AIF-deficient cells by scratch assay. High densities of cells were plated in replicate and allowed to attach for 12–36 h, and a scratch was made across the middle of each well with a P200 pipette tip. Scratch width was assessed immediately following cell displacement and 6–48 h later. AIF-deficient PANC-1, BxPC-3, and HPAC cells showed reduced migration while little change was observed in AIF-deficient HPAF-II or MIA PaCa-2 cells (Fig. 4b), in agreement with our proliferation rate data. It is notable that while MIA PaCa-2-shAIF cells displayed similar migration when compared to controls, when plated at the high densities used in the migration assay these cells took longer to adhere to plate surfaces. This suggests that AIF may be involved in cellular adhesion in this cell type; further studies are needed to define this function more clearly and determine the cancer specificity of this observation. Altogether, these data indicate that (1) the impact of AIF ablation upon pancreatic tumor cells is more severe than that observed in prostate cancer, (2) there is a spectrum of sensitivities to AIF ablation that is reflected by changes in cell growth patterns, and (3) AIF supports pancreatic tumorigenesis through a mechanism that appears different from that shown in prostate cancer.

### Cellular energy phenotype determines the ability of AIF to promote growth and survival of pancreatic cancer cells

**Fig. 5:**
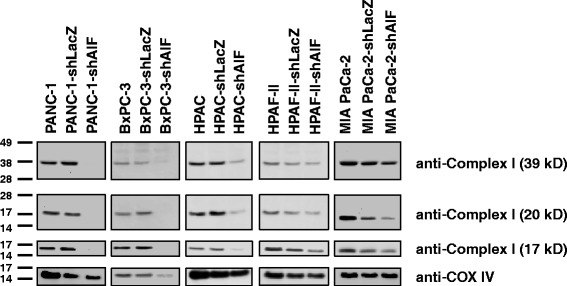
AIF selectively controls respiratory chain protein expression in pancreatic cancer cells. Following suppression of AIF, respiratory chain status was assessed by immunoblot analysis of complex I (39-, 20-, and 17-kDa subunits) and COX IV

Having found that AIF selectively contributes to the rates of both cellular proliferation and migration in vitro, we sought to determine how AIF supports cell growth in pancreatic cancer and distinguish these effects based on cellular metabolic state. A common feature of cells that require AIF for basal metabolic activity is a loss of expression in protein subunits of complex I of the respiratory chain [22, 28]. To determine whether respiratory chain regulation is related to AIF-mediated cell growth, cells were lysed and probed for complex I subunits by immunoblot analysis. Following knockdown of AIF the concomitant changes in respiratory chain protein levels were diverse and directly correlated with both metabolic phenotype and changes in growth. AIF-deficient PANC-1, BxPC-3, and HPAC cells exhibited substantial reductions in 39-kDa, 20-kDa, and 17-kDa complex I subunits (Fig. 5). Interestingly, when AIF was suppressed in BxPC-3 cells, the expression of not only complex I subunits but also COX IV was reduced (Fig. 5), a change that has not been previously reported in cancer and may suggest a more global alteration in the mitochondrial proteome in this cell type. Changes in respiratory chain status were minimal when AIF was depleted from HPAF-II and MIA PaCa-2 cells (Fig. 5). These data indicate that loss of complex I in pancreatic cancer cells following AIF ablation is dependent on metabolic phenotype.

**Fig. 6:**
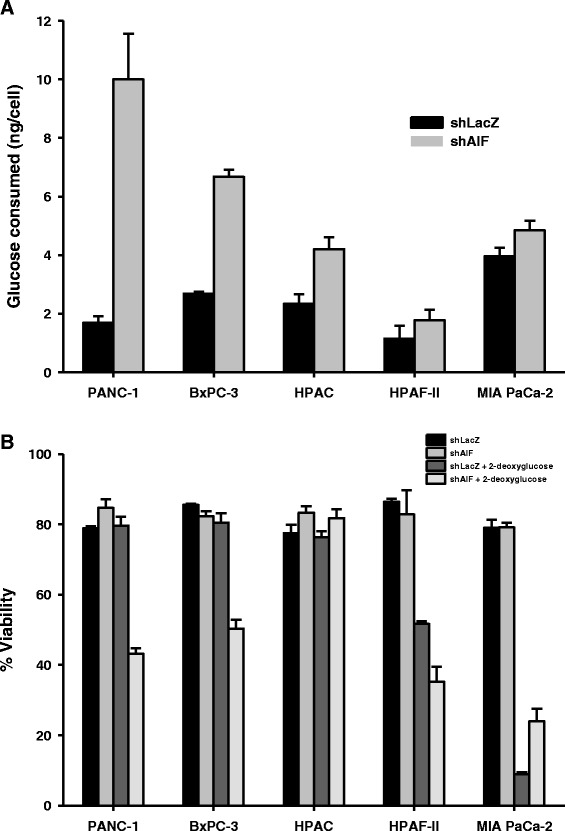
Glycolytic dependence predicts metabolic sensitivity to AIF ablation. *Glucose consumption assay* (Panel **a**): equal densities of cells were plated in fresh media, and total glucose was measured using the QuantiChrom™ Glucose Assay Kit (BioAssay Systems) 72 h after seeding. Total numbers of cells in each well were used to determine glucose consumption per cell. *Sensitivity to glycolytic disruption* (Panel **b**): Cells were seeded in equal densities and allowed to attach overnight before treatment with 50 mM 2-deoxyglucose. After 48 h cells were collected, and viability was determined by propidium iodide staining and flow cytometry. Data are shown as average ± standard deviation

To further define the role of AIF in pancreatic cancer cells, we next evaluated the metabolic changes associated with AIF ablation by measuring glucose consumption rates within our cell line panel. Increased glucose consumption is a common adaptation following impairment of the mitochondrial respiratory chain, allowing cells to meet ATP demands directly through glycolysis. In agreement with the spectrum of respiratory deficiencies we observed, AIF-deficient PANC-1, BxPC-3, and HPAC cells consumed ~2–5-fold more glucose than their corresponding controls, while HPAF-II-shAIF and MIA PaCa-2-shAIF cells exhibited glucose consumption levels that were essentially unchanged when compared to controls (Fig. 6a). Notably, the magnitudes of altered glucose consumption in our panel of cell lines directly correlated with the severity of respiratory chain deficiency that followed ablation of AIF (Fig. 5). This correlation suggests that differences in sensitivity to AIF ablation among cell types may stem from differential metabolic requirements prior to AIF ablation. For example, due to a pre-existing decrease in respiratory chain activity [63], MIA PaCa-2 and HPAF-II cells have already adapted by upregulating glycolysis such that further impairment of the respiratory chain via AIF ablation has no additional effects upon glucose consumption.

To test this hypothesis and to determine the benefit of AIF-mediated glucose metabolism to cell survival, we next inhibited glycolysis in our panel of cell lines by treatment with 2-deoxyglucose. Glycolytic cell lines (*i.e.*, those that rely on glycolysis rather than oxidative phosphorylation as a primary source of energy production) will exhibit a higher sensitivity to treatment than those that remain capable of using other pathways (such as lipid catabolism or glutaminolysis) to compensate for this metabolic deficiency. To assess sensitivity, cell viability was measured using propidium iodide staining followed by flow cytometry. Our results revealed that while control PANC-1 and BxPC-3 cells are entirely resistant to 2-deoxyglucose, those that lack AIF exhibit substantial sensitivity with only ~40–50 % survival following treatment (Fig. 6b). Taken together with the corresponding respiratory statuses of these cell lines (Fig. 5), AIF is likely to regulate a balance between glycolysis and oxidative phosphorylation that is critical to the growth and survival of PANC-1 and BxPC-3 cells. In contrast, AIF ablation did not affect sensitivity to treatment in HPAC cells despite their complex I deficiency and elevated glucose consumption levels (Fig. 6b). This is not surprising given the lipogenic nature of the HPAC cell line [53], which allows cells to circumvent the metabolic requirement for AIF and complex I but at the expense of their proliferative capacity (Fig. 4). When compared to the resistant PANC-1, BxPC-3, and HPAC cell lines, control HPAF-II and MIA PaCa-2 cells both displayed a high basal sensitivity to 2-deoxyglucose, reflecting their dependence on glycolysis that is likely due to long-term adaptations to basal mitochondrial dysfunction. AIF ablation modestly increased sensitivity to glycolytic disruption in HPAF-II cells, but MIA PaCa-2 cells displayed a pre-existing addiction to glycolysis [53, 64] that could not be amplified by AIF suppression. This sensitivity was comparable to PANC-1-shAIF and BxPC-3-shAIF cells following treatment (Fig. 6b) and is consistent with our glucose consumption data (Fig. 6a). In this context loss of AIF has little to no additional metabolic effect, an observation that further confirms the hypothesis that differences in glucose uptake among cell types following AIF ablation derive from differences in the intrinsic activities of their respiratory chains and/or glucose utilization.

### Matrigel™ growth conditions amplify AIF dependence in pancreatic cancer cells

**Fig. 7:**
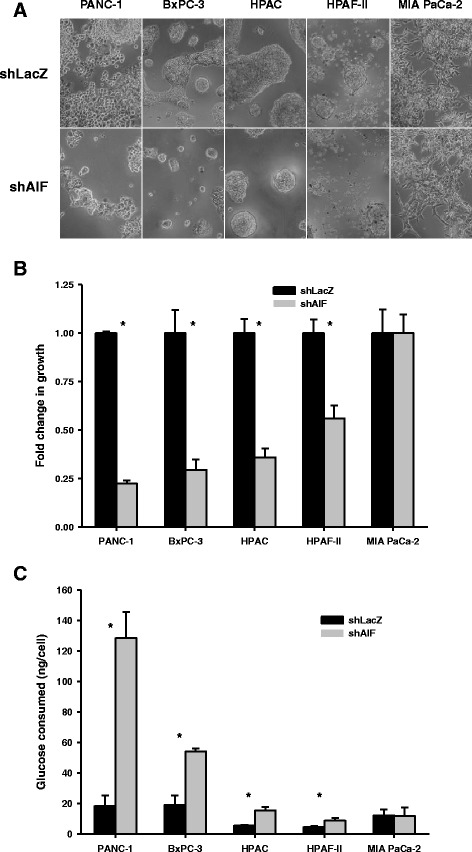
Matrigel™ environment amplifies dependence upon AIF-mediated growth and metabolism. *Matrigel™ growth*: Cells were plated in replicate Matrigel™ layers and allowed to grow for up to 21 days. Representative images (captured at 10× magnification) are shown (Panel **a**). Cells were extracted from substrate and quantified by Coulter™ counting (Panel **b**). *Glucose consumption in Matrigel™ substrate* (Panel **c**): Cells were exposed to Matrigel™ conditions prior to media collection and total glucose measurements using the QuantiChrom™ Glucose Assay Kit (BioAssay Systems). Cells were extracted from substrate with the Matrigel Recovery Solution (Corning) and quantified by Coulter™ counting. Total numbers of cells in each well were used to determine glucose consumption per cell. Data are shown as average ± standard deviation. *p < 0.05

Following the observations that AIF supports metabolism benefiting the growth and survival of non-glycolytic pancreatic cancer cells in vitro, we next explored the metabolic function of AIF in an environment that more closely resembles conditions found in vivo. Matrigel™ is a cell growth substrate that consists of matrix protein polymers and proteoglycans found in natural extracellular environments and is often used as a model for studying tumorigenesis in a setting that approximates in vivo cell growth and survival. To assess the role of AIF in the growth and metabolism of pancreatic cancer cells under such conditions, glucose consumption and growth were measured following exposure to Matrigel™ substrate. These results were strikingly similar to those found in the absence of Matrigel™, except that the differences between control and AIF-deficient cells were amplified. When introduced into substrate, cells displayed a dependence on AIF expression for aggressive growth and normal glucose consumption. While control PANC-1, BxPC-3, and HPAC cells grew into spheroidal tumor-like structures, those without AIF exhibited substantial reductions in both size (Fig. 7a) and proliferation rate (Fig. 7b). Furthermore, AIF-deficient PANC-1, BxPC-3, and HPAC cells consumed ~3–7-fold more glucose than those with AIF (Fig. 7c), suggesting that glycolysis becomes critical for growth and survival under Matrigel^TM^ growth conditions when AIF is depleted. In the HPAF-II cell line, which exhibited modest changes in growth and metabolism following AIF ablation in vitro, both shLacZ controls and shAIF failed to invade the substrate to the extent of other cell lines, yet HPAF-II-shAIF cells showed a substantial reduction in growth rate and a 2-fold increase in glucose consumption. Taken together with previous data, this suggests that AIF plays a minor role in HPAF-II glucose metabolism under nutrient-rich conditions but that this role gains prominence upon exposure to Matrigel^TM^. In contrast, MIA PaCa-2-derived cell lines did not display significant changes (Fig. 7), consistent with in vitro measurements. Following growth in Matrigel™ changes in both glucose uptake and growth in substrate were more severe but directly proportional to those found in vitro. Altogether, our data indicate that AIF supports the growth and survival of some pancreatic cancer cells by facilitating a metabolic balance, and this metabolic function is most beneficial to cell populations that do not rely fully on glycolysis for survival.

## Discussion

When functioning in a pro-death role, AIF can undergo nuclear translocation followed by the induction of chromatin condensation and DNA degradation during various forms of cell death [65–69]. As a promoter of caspase-independent death, it is formally possible that AIF could act in a tumor-suppressive manner. Yet while AIF nuclear translocation has been observed in cancer [70, 71], this study and others [28, 29] show that loss of AIF suppresses tumorigenesis and that AIF’s nuclear function is unlikely to make a significant contribution to death pathways despite overexpression in tumors [28, 30–35]. AIF elevation in cancer is modest (typically less than 3-fold), suggesting a threshold exists above which AIF expression is either no longer beneficial or actively disadvantageous. Despite this threshold, AIF elevation in cancer is both sufficient and necessary for AIF to promote survival through its enzymatic activity [28, 29].

Presently, AIF’s enzymatic activity has been demonstrated to support tumorigenesis through at least two distinct mechanisms. In colorectal cancer cells, AIF elevates the cellular oxidative state to protect against chemical stress-induced apoptosis [29]. In prostate cancer, AIF promotes a metabolic state that selectively supports the growth and survival of cells that have achieved advanced status [28], suggesting that AIF addiction in cancer manifests as tumors become increasingly aggressive. Our current data agree with a similarly important contribution for AIF to pancreatic cancer progression, a disease setting that most often presents at an advanced stage by the time of diagnosis. However, in contrast to our observations in prostate cancer, the selectivity of AIF’s support of different pancreatic cancer cell types was directly related to their cellular energy preferences.

PANC-1, BxPC-3, and HPAC cells, all of which display a metabolic phenotype not solely reliant upon glycolysis (Fig. 6b, Table 1, [53]), exhibited a remarkably similar reduced growth phenotype (~60 % of controls) following AIF ablation that also included respiratory chain depletion and elevated glucose consumption levels ranging from ~2–5-fold. When introduced into Matrigel™ substrate, we observed more drastic changes in both growth (20–30 % of controls) and glucose uptake (~3–7-fold increases). PANC-1 and BxPC-3 cell lines were also sensitized to glycolytic disruption, suggesting that glycolysis becomes critical for survival following AIF ablation. This change was not observed in the HPAC cell line, likely due to a metabolic flexibility that derives from its lipogenic phenotype [53]; in this context AIF supports aggressive growth, yet cells remain capable of maintaining survival in the absence of AIF’s metabolic activities.

HPAF-II cells, which exhibit a greater dependence on glycolysis than PANC-1, BxPC-3, or HPAC cells (Fig. 6b, Table 1, [53]), also showed changes following AIF ablation, although less severe. Cells did not lose complex I subunits, nor did they exhibit significantly increased glucose consumption in vitro. Despite this, AIF ablation further increased sensitivity to 2-deoxyglucose while modestly compromising in vitro growth. Moreover, introduction to Matrigel™ caused HPAF-II-shAIF cells to elevate glucose consumption by 2-fold while significantly reducing growth rate. No changes in either growth or glucose metabolism were identified in MIA PaCa-2 cells, which displayed a severe pre-existing addiction to glycolysis (Fig. 6b, Table 1, [53]) that could not be further exacerbated by AIF ablation.

While our data suggest that AIF is selectively beneficial to metabolically “flexible” PDAC cells that use both glycolysis and oxidative phosphorylation for energy production, the basis for the selective sensitivity of different cell types to AIF ablation has not been firmly established. It has been proposed that AIF functions as a metabolic sensor through binding and oxidizing NADH ligands [25]. In light of this hypothesis and extending our current data to other cell types, AIF may regulate respiratory chain expression and metabolic flux in response to NADH/NAD^+^ availability (established by the overall metabolic state). By implication, AIF is consequently central to a self-regulating metabolic balance.

How might the presence of AIF impact metabolism addiction in pancreatic cancer? Otto Warburg first observed that cancer cells increase their glucose consumption levels relative to normal cells, hypothesizing that defective mitochondrial respiration contributes to tumorigenesis [72]. While elevated glucose consumption is a common characteristic of tumor cells, it is now well-established that many cancer cells rely on both glycolysis and mitochondrial energy metabolism to coordinate an efficient balance between glucose-derived macromolecule biosynthesis and energy production that permits aggressive growth and survival [73]. The supportive role of AIF in cancer derives from its ability to maintain efficient mitochondrial electron transport and oxidative phosphorylation, and this function becomes critical as cells become more aggressive and reliant on mitochondrial function. Indeed, several recent studies have identified paramount roles for oxidative phosphorylation in promoting the invasiveness of cancer cells [38, 74, 75]. When tumors undergoing aerobic glycolysis reach advanced stages, cancer cells often continue to rely on mitochondria as a source of energy production [76] and suffer a metabolic disadvantage when mitochondrial function is lost. In pancreatic cancer, this is especially true. Recently it has been shown that following ablation of the oncogene KRAS, mitochondrial function and oxidative phosphorylation become critical for survival in relapsing tumors [38]. Our data suggest that AIF expression is necessary to mediate a metabolic balance in certain pancreatic cancer cells (e.g., PANC-1, BxPC-3, and HPAC), and AIF ablation induces cellular adaptations that lead to a greater reliance upon glycolysis for survival in these cell types. Other cell types that exhibit a pre-existing addiction to glycolysis (e.g., HPAF-II and MIA PaCa-2) are therefore less sensitive to AIF ablation. This further emphasizes the impact of metabolic balance in support of pancreatic tumorigenesis, specifically through the expression of AIF.

In this study we established a pro-tumorigenic role for AIF in pancreatic cancer that derives from its metabolic activities. The selectivity of AIF dependence in tumorigenesis appears independent of oncogene/tumor suppressor status and instead stems from the overall cellular metabolic state, which results from the cumulative effect of genetic alterations. We found a direct relationship between basal metabolic state and dependence upon AIF expression (Table 1): as cells become aggressive such that they require a critical balance between mitochondrial energy metabolism and glycolysis, they become more dependent upon AIF. This correlation is in strong agreement with other studies. For example, PANC-1 cells (highly sensitive to AIF ablation) express higher levels of vascular endothelial growth factor than MIA PaCa-2 cells (insensitive to AIF ablation) [59, 77, 78]. This indicates a greater requirement for oxygen and mitochondrial function, and hence AIF activity, in the PANC-1 line. A recent report identified clear PDAC subtypes based upon metabolic requirements [53]. This extensive study characterized the metabolic states of all cell lines used in our study (Table 1) as well as numerous other cell lines, and their data strongly support our model. These data and our previous studies suggest that AIF’s metabolic impact upon tumorigenesis increases with disease progression. Previously we showed that AIF promotes a metabolic state permitting progression to advanced stages. Based on our current data, we propose that in the most advanced tumor cells (such as in PDAC), which critically rely on metabolic reprogramming for growth and survival, AIF activity is maximally exploited and becomes entwined within the overall metabolic state, a situation in which the only limiting factor to AIF dependence is how the cell utilizes mitochondrial energy metabolism.

Altered AIF expression is not a general feature of cancerous tissues and AIF activity is not universally supportive to cancer development and progression. However, in studies presented here we have identified a subpopulation of pancreatic cancer samples in which AIF expression is elevated, and using a panel of cancer cell lines with defined metabolic phenotypes we have correlated AIF activity to basal metabolic state. At present the molecular mechanisms defining AIF sensitivity remain to be elucidated, yet the experiments presented outline a framework for determining those cells in which AIF activity is critical, based on criteria such as metabolic phenotype and fuel source preference/requirements. The promise of this framework is the potential for AIF-mediated therapy. Development of such therapy, either as a stand-alone approach or more likely in combination with other modalities, will depend on better understanding of AIF mechanism and accurate metabolic assessment, but offers significant potential for increasing our treatment arsenal for cancer patients suffering from advanced disease.

## Conclusions

Altogether this study highlights the metabolic significance of AIF to PDAC and expands the range of AIF function in tumorigenesis. We found that the basal energetic requirements of PDAC cells determine the ability of AIF to support metabolic plasticity that benefits growth and survival. As a metabolic linchpin in cancer, AIF therefore represents a novel therapeutic target.

### Ethics statement

All work performed in this study was carried out in accordance with the Declaration of Helsinki. Patient-derived gene expression data was obtained from the publically available database Oncomine, which requires written informed consent and institutional review board approval from all investigators prior to data deposition. All cell lines used in this study were obtained from commercial sources; no other clinical specimens or human subjects were employed.

## Abbreviations

AIF: apoptosis-inducing factor
FBS: fetal bovine serum
PBS: phosphate buffered saline
RNAi: RNA interference
shRNA: short-hairpin RNA
